# The Controversial Role of Retinoic Acid in Fibrotic Diseases: Analysis of Involved Signaling Pathways

**DOI:** 10.3390/ijms14010226

**Published:** 2012-12-21

**Authors:** Tian-Biao Zhou, Gregor P. C. Drummen, Yuan-Han Qin

**Affiliations:** 1 Department of Pediatric Nephrology, the First Affiliated Hospital of Guangxi Medical University, Nanning 530021, China; E-Mail: a126tianbiao@126.com; 2 Cellular Stress and Ageing Program, Bionanoscience and Bio-Imaging Program, Bio & Nano-Solutions, Helmutstr. 3A, Düsseldorf 40472, Germany; E-Mail: gpcdrummen@bionano-solutions.de

**Keywords:** fibrotic disease, fibrosis, retinoic acid, extracellular matrix, signaling pathways

## Abstract

Fibrotic diseases, such as liver, pulmonary and renal fibrosis, are common end-stage conditions and represent a major global health problem. Furthermore, effective therapeutic measures are presently unavailable. Extracellular matrix accumulation is the most prominent characteristic in the pathogenesis of fibrotic disease. Retinoic acid, including all-*trans* retinoic acid, 9-*cis* and 13-*cis* retinoic acid, play important roles in various physiological processes, such as in embryonic development, reproduction, vision, cell growth, differentiation, apoptosis and inflammation. Present studies report that retinoic acid treatment may affect various processes involved in the onset and progression of fibrotic disease. However, the therapeutic effects of retinoic acid in such diseases remain controversial. Several reports indicate that retinoic acid positively affects the progression of fibrosis and alleviates the accumulation of the extracellular matrix, whereas other studies report the opposite; that retinoic acid exacerbates fibrosis and induces extracellular matrix accumulation. Signaling pathways might be an important influencing factor and differences in signaling events might be responsible for the contradictory role of retinoic acid in fibrotic diseases. Since there was no review available that investigated the role of retinoic acid and the signaling pathways involved, we retrospectively studied the literature and provide a comprehensive analysis of retinoic acid’s role in fibrotic diseases, and provide an overview of the signal transduction pathways involved in its pathogenesis.

## 1. Introduction

Fibrotic diseases, such as liver, pulmonary, and renal fibrosis, are common end-stage conditions that are major health problems worldwide and put significant strain on the health-care systems of numerous countries. Commonly, fibrotic diseases are characterized by the formation of excess fibrous connective tissue, which significantly impairs the organ’s ability to function normally. Liver fibrosis is a common end-stage condition of many chronic liver diseases that occurs after incomplete recovery from hepatocyte damage. During fibrosis progression, hepatocellular damage and inflammation trigger complex cellular events that result in collagen deposition and the disruption of the normal liver architecture [1], culminating in cirrhosis, portal hypertension, and liver failure. Pulmonary fibrosis (e.g., idiopathic pulmonary fibrosis and cystic fibrosis) is a progressive disease with largely unknown etiology, and is characterized by extensive remodeling of the lung parenchyma, ultimately resulting in respiratory failure [2]. Renal fibrosis is the inevitable consequence of the synthesis and excessive deposition of extracellular matrix (ECM) within the glomerulus and interstitium [3]. The prognosis in fibrotic disease is generally poor, regardless of the organ involved and currently, no effective therapies are available or remain at the experimental stage.

ECM accumulation is the most important characteristic in the pathogenesis of fibrotic diseases. ECM is composed of a cross-linked porous network of multifibril collagens and glycosaminoglycans, including various collagens, fibronectin (FN), laminins (LN) and thrombospondin (TSP), *etc.* [4–6]. One of the primary functions of the ECM is to maintain tissue integrity and homeostasis, and to provide a reservoir of cytokines and growth factors [7]. Maintaining the ECM’s homeostasis is therefore highly important to preserve the normal physiologic function of cells and tissues; underexpression of ECM components induces the collapse of cells/tissues, whilst the accumulation of ECM provokes the progression of fibrosis. The development of focused therapeutic interventions for diseases in which the maintenance of the ECM’s homeostasis goes awry is highly important to combat fibrotic pathophysiology and to improve disease prognosis and outcome.

Retinoic acid (RA), an active metabolite of vitamin A, belongs to the retinoid family and includes the isoforms all-*trans* retinoic acid (ATRA), 9-*cis* retinoic acid (9-*cis*-RA), and 13-*cis* retinoic acid (13-*cis*-RA), *etc*. (Table 1). RA is involved in various physiological processes, such as embryonic development, reproduction, vision, cell growth, differentiation, apoptosis, and inflammation [8–10]. Vitamin A deficiency can lead to the increased expression of FN, LN and collagen IV [11]. Furthermore, various studies previously reported that RA regulates the expression of ECM and plays a significant role in fibrotic diseases. However, the effects of RA on ECM accumulation and fibrosis are controversial. Several studies found that RA reduced the expression of ECM, whilst other reports show quite the opposite; that RA induces ECM accumulation. Concomitantly, several studies found that RA had a protective and positive effect on fibrosis, but equally reports can be found in the literature that show that RA exacerbated the fibrotic disease.

Because such divergent and contradictory effects are reported in the literature, we hypothesize that these effects might be caused by the fact that different signaling transduction pathways might be involved in various tissues and/or cells. Furthermore, the different RA doses used in various studies might be another reason for the contradictory results reported for RA treatment in fibrotic disease.

To the best of our knowledge, no comprehensive review on the role of RA in ECM metabolism and fibrotic disease, and the signaling pathways involved is currently available; this despite the fact that an inventory of these pathways is urgently necessary in order to develop new research angles. We retrospectively studied the literature to provide a comprehensive analysis of retinoic acid’s role in fibrotic diseases, and in this review provide an overview of the signal transduction pathways involved in its pathogenesis.

## 2. Role of RA in Diseases

### 2.1. Role of RA in Liver Fibrosis

Liver fibrosis is a common feature and end-stage condition of many chronic liver diseases with various etiologies. As the main complication of chronic liver damage, liver fibrosis is essentially a wound healing process characterized by the accumulation of ECM proteins in the liver [12,13] that leads to scarring and impairment of liver function. Several studies found that RA may play a protective role against ECM accumulation and liver fibrosis *in vivo*. Wang L *et al.* [14] found that ATRA reduced the amount of histological detectable fibrosis induced by carbon tetrachloride (CCl_4_) in C57BL/6J mice, and this was accompanied by an attenuation of the accumulation of collagen α2 (I). That ATRA ameliorates CCl_4_-induced liver fibrosis was confirmed by Hisamori *et al.* [15] in BALB/c mice. Equally, Yang KL *et al.* [16] reported that a RA derivative isolated from the mycelium of *Phellinus linteus* could antagonize ECM accumulation and liver fibrosis in BALB/c mice by down-regulating reactive oxygen species (ROS) generation and calcium influx, thereby directly affecting transforming growth factor-β1 (TGF-β1). Wang H and co-workers [17] showed that ATRA (1.5 and 7.5 mg/kg) was able to inhibit common bile duct ligation (CBDL)-induced liver fibrosis in female Wistar rats, and ATRA reduced the expression of collagen I protein more greatly than that of 0.1 mg/kg. The expression of type I collagen (COL-I), tissue inhibitors of metalloproteinase-1 (TIMP-1), TGF-β1, and connective tissue growth factor (CTGF) were reduced, whilst He H *et al.* [18] reported that 5 mg/kg ATRA significantly reduced liver fibrosis and nearly eliminated liver necrosis after CBDL in male Sprague-Dawley rats, especially in combination with ursodeoxycholic acid (UDCA). Most importantly, they found that ATRA alone or in combination with UDCA repressed CYP7A1 expression in human hepatocytes, and significantly inhibited collagen 1A1 (COL-1A1), matrix metalloproteinase-2 (MMP-2), and α-smooth muscle actin (α-SMA) expression and/or activity in primary human hepatic stellate cells (HSCs) and LX-2 cells (a HSC cell line), and TGF-β1 induced Smad2 phosphorylation in LX-2 cells.

*In vitro*, a number of studies also found that RA could play a protective role against ECM accumulation. For instance, Hellemans *et al.* [19] reported that ATRA exerted a significant inhibitory effect on the synthesis of procollagen I, III, and IV, FN, and LN in HSCs. Conversely, 9-*cis*-RA increased procollagen I, but did not affect the expression of other matrix proteins. Recently, Ye Y *et al.* [20] reported that ATRA inhibits proliferation and collagen production in HSCs via attenuation of the mRNA expression of collagene genes, *i.e*., procollagen α1 (I) and α1 (III), and the profibrogenic genes TGF-β1, CTGF, MMP-2, TIMP-1, TIMP-2, and plasminogen activator inhibitor-1 (PAI-1). Concurrently, the mRNA expression of MMP-3 and MMP-13 was stimulated by suppression of c-Jun *N*-terminal kinase (JNK) and active protein-1 (AP-1). The notion that RA and its derivatives could prevent ECM accumulation in HSCs was further corroborated by Yang K *et al.* [16] and He H and collaborators [18]. The latter group reported that 5 μM ATRA significantly suppressed COL-1A1 mRNA expression by more than 50% in HSCs [18]. Hisamori S *et al.* [15] performed a study in HSCs and disclosed that the administration of ATRA down-regulated the production of TGF-β1, interleukin-6 (IL-6), collagen, nuclear factor-κB p65, and p38 mitogen activated protein kinase (p38MAPK). Radaeva *et al.* [21] showed that in early activated HSCs compared with quiescent or fully activated HSCs, RA and retinal dehydrogenase (Raldh) levels were upregulated. They also showed that blocking RA synthesis with a Raldh inhibitor or a retinoic acid receptor antagonist abolished the up-regulation of retinoic acid early inducible gene 1 (RAE-1), which in a chronic status would lead to chronic liver fibrosis. Conversely, treatment with RA or oxidation of retinol to RA induced RAE-1 expression in HSCs, and sensitizes early activated HSCs to NK cell killing.

Despite the aforementioned results, three reports suggest that quite the opposite occurs and that RA enhances ECM production and exacerbates liver fibrosis both *in vitro* and *in vivo*. Interestingly, those three reports from the same study group and thus lack independent confirmation. Okuno *et al.* [22] reported that 9-*cis*-RA exacerbated rat liver fibrosis by inducing the activation of TGF-β1 in male Wistar rats. However, the used dose in their study was markedly higher compared with the doses used in other *in vivo* studies (40 mg/kg of body weight, 5 times a week; see Table 2). They also performed a study *in vitro* using HSCs, and found that RA enhanced plasminogen activator (PA)/plasmin levels and thereby induced proteolytic activation of TGF-β1, a strong fibrogenic cytokine, resulting in enhanced ECM production [22,23].

In agreement with the majority of studies mentioned above, Ye *et al*. found that a lower dose of RA could inhibit the expression of TGF-β1 and could play a protective role against cell injury, proliferation, and collagen accumulation [20]. However, at higher doses, Okuno showed that RA up-regulated the expression of TGF-β1 and negatively influenced ECM accumulation and liver fibrosis [22]. There might be a common pathway in which RA displays a protective effect or an adverse effect, which is obviously dose dependent.

The characteristics of the studies mentioned above in which the effect of RA on liver fibrosis were evaluated are summarized in Table 2, and we found that the used dose of RA in the study by Okuno *et al.* [22] was significantly higher than in other studies, especially in the animal experiments.

### 2.2. Role of RA in Pulmonary Fibrosis

Pulmonary fibrosis is an intractable paranchymal lung disease that is characterized by persistent alveolitis and accumulation of connective tissue and ECM [24]. The pathogenesis of pulmonary fibrosis is not well understood, but the occurrence of fibrotic lesions is often due to secondary disease causes or irritants, e.g., viral infections, sarcoidosis, systemic sclerosis, *etc.*, long-term exposure to occupational and/or environmental inhalants, radiation from cancer treatment, certain drugs, or idiopathic in origin. The currently available *in vitro* and *in vivo* reports indicate that RA plays a protective role and prevents ECM accumulation in the lung, thereby protecting against pulmonary fibrosis. In C57BL/6 female mice, Tabata *et al.* [25] demonstrated that ATRA prevented radiation- or bleomycin-induced pulmonary fibrosis. They found that ATRA reduced irradiation-induced IL-6 production through the protein kinase C (PKC)-δ/NF-κB pathway, and inhibited irradiation-induced TGF-β1 production through the p38MAPK/NF-κB pathway, resulting in the inhibition of cell differentiation and collagen synthesis. Esteban-Pretel *et al.* [26] found that RA could reduce the amount of collagen IV and down-regulate the expression of proinflammatory cytokines, interleukin-1α (IL-1α), interleukin-1β (IL-1β) and tumor necrosis factor-α (TNF-α) in vitamin A-deficient rats. However, they also recorded that RA treatment increased oxidative stress and lipid peroxidation, most likely because of auto-oxidation of RA due to the high *p*O_2_ in the lung that produced superoxide and carbon-centered radicals.

Ozer *et al.* [27] reported that the degree of fibrosis and α-SMA expression showed a significant decrease in RA-treated newborn Wistar rats with oxygen-induced lung injury. Tabata *et al.* [28] found that ATRA could reduce IL-6 expression and concomitantly NF-κB, blocked IL-6R and gp130, and likely reduced radiation-induced pulmonary fibrosis via a PKCδ-dependent pathway. Dong Z and co-workers [29] recently showed that ATRA is able to decrease the expression of interleukin-17A (IL-17A), IL-6, and TGF-β1, and could alleviate bleomycin-induced pulmonary fibrosis in C57BL/6 male mice. *In vitro*, Tabata C *et al.* [25] found that ATRA reduced irradiation-induced production of IL-6, TGF-β1, α-SMA and COL-1A1 in lung fibroblasts. Tabata C and collaborators [28] also found that ATRA reduced the activated form of NF-κB p65 and reduced the expression of IL-6 in lung fibroblasts.

All studies examined, report positive effects on pulmonary fibrosis under treatment with RA. The characteristics of the studies mentioned above evaluating the effect of RA on pulmonary fibrosis are summarized in Table 3.

### 2.3. Role of RA in Kidney Fibrosis

Kidney fibrosis is a major and final pathological manifestation of progressive kidney disease and is characterized by the activation of fibroblasts, epithelial-to-mesenchymal transition, monocyte and/or macrophage infiltration, and excessive ECM accumulation along with a loss of functioning nephrons [30]. This contributes to a progressive decrease in glomerular filtration and tubular function. Numerous studies performed *in cellulo* and animal models report that ATRA is able to alleviate glomerulonephritis and renal interstitium disease.

From *in vivo* studies, Wagner *et al.* [31] reported that both ATRA and 13-*cis*-RA significantly reduced glomerular α-SMA and alleviated glomerular proliferation, glomerular lesions, and albuminuria in a Thy1.1-induced mesangioproliferative glomerulonephritis rat model. In continued investigations by the same group, Morath *et al.* [32] went on to show that the beneficial effects of the retinoids, in particular ATRA, on glomerular damage could be attributed to a distinct reduction in renal TGF-β1 and TGF receptor II (TGFRII) expression. Lehrke *et al.* [33] reported that treatment of glomerulonephritic rats with agonists specific for the retinoid X receptor (Ro-257386) completely inhibited induction of TGF-β1, procollagen I, and FN expression in Thy1.1-induced glomerulonephritis rats. In turn, Oseto *et al.* [34] found that ATRA also significantly reduced proliferating cell nuclear antigen (PCNA), ED-1, α-SMA, TNF-α, IL-1β, and CCAAT enhancer-binding protein 8 (C/EBP8) in glomerulonephritis rats. Furthermore, down-regulation of the cell proliferation-related platelet derived growth factor (PDGF) and fibrosis-related TGF-β1, and COL-1 were recorded. Schaier *et al.* [35] reported that both the retinoic acid receptor alpha (RARα-specific agonist AGN 195183 and the retinoid X receptor (RXR) specific agonist AGN 194204 reduced the gene expression of glomerular TGF-β1 and prepro-ET1, and alleviated renal damage in rats with established chronic glomerulonephritis (chronic mesangioproliferative Thy-GN (MoAb 1-22-3) rat model). Adams *et al.* [36] found that 13-*cis*-RA acted as a potent immunosuppressive and antifibrotic agent able to prevent and inhibit the progression of glomerulosclerosis and interstitial fibrosis in a chronic Fisher344→Lewis kidney allograft rat model. The mRNA expression of the chemokines monocyte chemotactic protein-1/chemokine (C-C motif) ligand 2 (MCP-1/CCL2), macrophage inflammatory protein-1α/chemokine (C-C motif) ligand 3 (MIP-1α/CCL3), interferon gamma-induced protein 10/C-X-C motif chemokine 10 (IP-10/CXCL10), and Regulated And Normal T cell Expressed and Secreted/chemokine (C-C motif) ligand 5 (RANTES/CCL5), and proteins associated with fibrosis, *i.e*., PAI-1, TGF-β1, and collagens I and III, were strikingly lower in treated allografts. Recently, He *et al.* [37] established that ATRA inhibited cell proliferation and induces differentiation in HIV-infected podocytes through RARα-mediated cAMP/PKA activation, whilst in HIV-1-transgenic mice they recorded reduced proteinuria, cell proliferation, and glomerulosclerosis. Xu *et al.* [38] performed a highly interesting study using three doses of ATRA (low doses, 6–10.7 mg/kg/day; medium doses, 12.7–18.8 mg/kg/day; high doses, 20.1–27.4 mg/kg/day) in Alb/TGF-β1 transgenic mice (induces progressive renal fibrosis and retinoic acid deficiency in the kidneys), and reported that the low doses had a tendency to reduce the average urinary albumin excretion, as well as renal retinaldehyde dehydrogenase 2 (RALDH2; catalyzes the synthesis of RA from retinaldehyde), FN, COL-1A1, COL-1A2 and COL-4A1 compared with the control group (C57BL/6J × CBA F1 mice). Nonetheless, the extent of renal fibrosis remained unchanged. Most importantly, with increasing dose, reduction of the aforementioned factors disappeared, mortality rates increased, and RALDH2 and connective tissue growth factor (CTGF) mRNAs significantly increased. This study underscores the Janus-like properties of RA, and therefore further studies are desperately needed to determine where RA’s profibrotic and toxic action begins and ends. *In vitro*, Adams *et al.* [36] performed a study in peritoneal macrophages, and found that 13-*cis*-RA could pronouncedly decrease the protein secretion of inflammatory cytokines (TNF-α, IL-6, IL-1β, IL-10).

In human mesangial cells, Wen *et al.* [39] reported that 9-*cis*-RA attenuated TGF-β1-induced α-SMA, FN, and PAI-1 expression, but it did not significantly affect cell proliferation and survival. As the authors express it, it is interesting that part of their study shows that 9-*cis*-RA induced hepatocyte growth factor (HGF) mRNA expression and protein secretion, stimulated HGF promoter activity, activated c-met receptor phosphorylation, and induced expression of the Smad transcriptional co-repressor TGIF. In an overexpression approach of TGIF or treatment with 9-*cis*-RA resulted in suppression of trans-activation of the TGF-β-responsive promoter and conditional ablation of the c-met receptor completely abolished the anti-fibrotic effect of 9-*cis*-RA and abrogated TGIF induction. Collectively, the results of these studies suggest that 9-*cis*-RA exerts its anti-fibrotic action by antagonizing TGF-β1 and that this is possibly mediated via a HGF/c-met receptor signaling mechanism. Liu and co-workers [40] found that treatment with two different doses of ATRA (5 mg/kg/day and 10 mg/kg/day by gastric gavage) lowed the glomerulosclerosis index and increased renal function in 5/6 nephrectomized Sprague Dawley rats, which was at least partly attributable to a reduction in PAI-1 and α-SMA expression; plasmin and MMP-2 levels remained unchanged. Kishimoto *et al.* [41] equally showed that ATRA could significantly improve the histological and immunological overall image, including the extent of macrophage infiltration and could improve the expression of MCP-1, TGF-β1, α-SMA and collagen I in unilaterally ureterally obstructed C57BL/B6 mice. Importantly, the authors determined in preliminary experiments that ATRA induced apoptosis, predominantly in the infiltrating cells, which might be an important reason for the improvement in renal interstitial fibrosis. Mallipattu *et al.* [42] found that ATRA could up-regulate the gene expression of GSTA4, PER2, IFRD1, HIVEP1, LIPE, METTL1, RFX1, SNX5, PLAUR, CLCN3, SFXN4, DMD, FOXC1, KLF15, GDNF, GABARAPL1 and could down-regulate the gene expressions of GATA3, CCND2, ISGF3G, MCAM, SIAT1, FGF18, PITX2, GNB4, WISP1, YWHAZ, CACNA1C in cultured podocytes. Our previous studies [10,43,44] distinctly showed that ATRA was able to increase the expression of MMP-2 and MMP-9, and reduce the expression of apoE, Col-IV, FN, and TGF-β1 in glomerulosclerosis rats. And we also found in renal interstitial fibrosis rats that the expression of prohibitin was increased by ATRA (15 mg/kg/day).

However, despite the numerous reports that demonstrate positive effects on renal fibrosis, a few studies reported that ATRA treatment exacerbated the glomerulonephritis and renal interstitium disease. For instance, Iyoda *et al.* [45] found that the renal pathology in cryoglobulin-associated membranoproliferative glomerulonephritis mice (mice overexpressing thymic stromal lymphopoietin [TSLP]) was aggravated after treatment with ATRA (20 mg/kg 3 times weekly by intraperitoneal injection), and significantly increased glomerular collagen IV in cryoglobulin-associated membranoproliferative glomerulonephritis mice at 4 weeks but not 8 weeks. Beside these changes, the authors reported systemic deterioration and unexpected changes in immunoglobulins. They propose that the negative effects induced by ATRA might be an immunomodulatory effect of ATRA on B cell function. Moulder *et al.* [46] reported that ATRA exacerbated radiation nephropathy in rats and they speculated that this negative effect might be the result of stimulation of renal cell proliferation or inhibition of renal nitric oxide activity. Likewise, Alique and coworkers [47] demonstrated that ATRA exacerbated glycated albumin effects on intracellular oxidation and the expression of the molecules involved in leucocyte infiltration in cultured human mesangial cells. In patients treated with ATRA for acute promyelocytic leukemia, retinoic acid syndrome (RAS), which is characterized by an inflammatory reaction with capillary leakage and myeloid cell tissue invasion that presents with cardiopulmonary symptoms and occasionally acute kidney injury, is a serious and documented complication [48]. Such studies suggest that caution should be exercised when translating ATRA treatment of human disease based on the largely positive animal data reported in the literature.

The characteristics of the studies that evaluated the effect of RA on kidney fibrosis discussed here are summarized in Table 4.

### 2.4. Role of RA in Other Fibrosis

The role that RA plays in fibrosis of various other tissues and organs, and the signaling pathways involved are equally diverse as in the aforementioned organs. It becomes clear that not only signaling with respect to constituents of the ECM, but also mediators or inhibitors of inflammation, oxidative stress and others are involved.

Choudhary *et al.* [49] reported that ATRA could significantly reduce interstitial and perivascular fibrosis in aortic restricted Sprague-Dawley rats. RA inhibited the cleavage of caspase-3 and-9, restored the ratio of Bcl-2 to Bax, and prevented a decrease in SOD-1 and SOD-2 levels in pressure-overloaded rats. Furthermore, the authors state that pressure overload-induced phosphorylation of ERK1/2, JNK, and p38 was inhibited by RA via up-regulation of mitogen-activated protein kinase phosphatase 1 and 2 (MKP-1; MKP-2). The pressure overload-induced production of angiotensin II was attenuated by RA via up-regulation of angiotensin-converting enzyme 2 (ACE 2) and through the inhibition of the expression of cardiac and renal renin, angiotensinogen, ACE1, and angiotensin type 1 receptor (AGT1R). Klopcic and co-workers [50] found that ATRA reduced inflammation, tissue destruction, and fibrosis in a 2,4,6-Trinitrobenzene Sulfonic Acid (TNBS)-induced intestinal fibrosis mice model, whilst indomethacin increased TNBS-induced fibrosis. The authors also suggest that the reported opposing effects may be linked via secreted protein acidic and rich in cysteine (SPARC); an acidic ECM glycoprotein that plays an important role in cell-matrix interactions, collagen binding, and bone mineralization.

Okoshi *et al.*’s [51] research shows that administration of ATRA attenuated irradiation-induced intestinal fibrosis and concomitantly reduced the expression of IL-6 and TGF-β1. They could determine from further *in vitro* studies that ATRA suppressed the trans-differentiation of irradiated intestinal fibroblasts and reduced the production of α-SMA and collagen. Wang *et al.* [52] reported that ATRA could reduce the expressions of TGF-β1 and collagen I in peritoneum tissue and could prevent peritoneal fibrosis in rat model of peritoneal dialysis. Treharne *et al.* [53] found that RA could reduce the extent of the fibrotic lesions in cystic fibrosis, and could increase the expression of tissue transglutaminase (Tgase2); Tgase2 crosslinks proteins between ɛ-amino groups a lysine residues and γ-carboxamide groups of glutamine residues (increased stability and resistance to proteolysis) and also acts as a G-protein. In turn, Tgase2 induced the expression of nucleoside diphosphate kinase (NDPK), a membrane-bound protein kinase that catalyzes the exchange of phosphate groups between different nucleosides and is known to control G-protein function.

**Figure 1:**
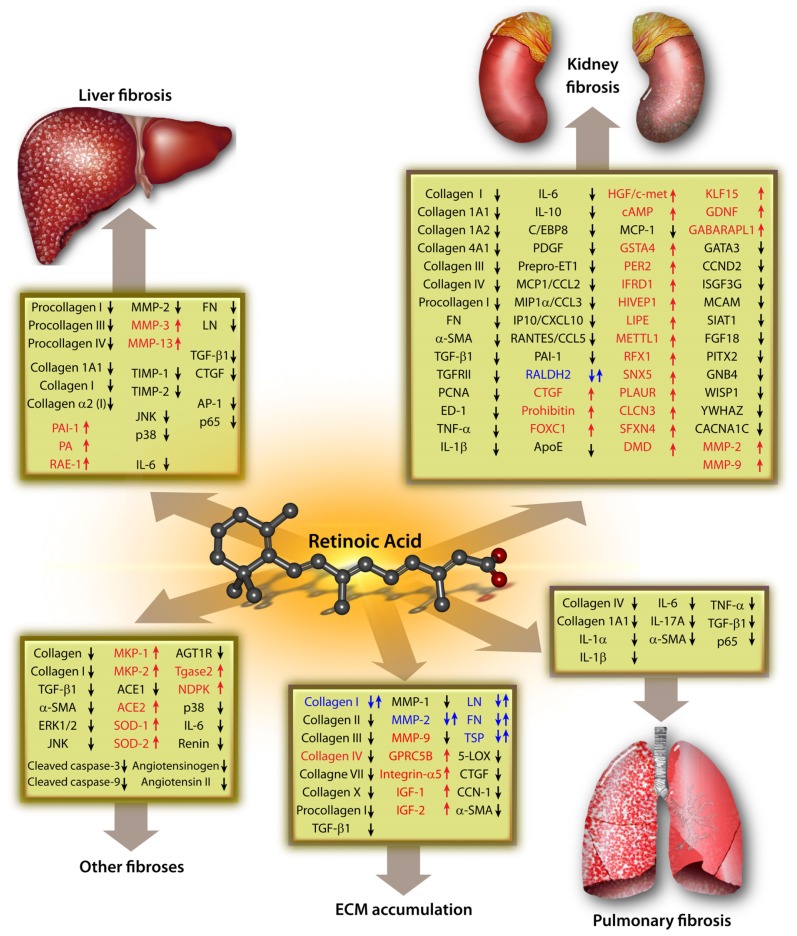
Signaling pathways that are affected by retinoic acid in various fibrotic diseases. Note: ↑ = stimulation; ↓ = inhibition; ↑↓ = both stimulation and inhibition have been reported.

The signaling pathways for RA in miscellaneous fibrosis are summarized in Figure 1.

## 3. Signaling Pathways of Role of RA in the ECM Metabolism

RA inhibits the expression of TSP [4], TGF-β1 [54], collagen I [54–58], collagen II [59], collagen III [54,58], collagen VII [58], collagen X [59], LN [55,60], α-SMA [55], FN [4,55,56,58,61,62], 5-lipoxygenase (5-LOX) [54], CTGF [54], cysteine-rich protein 61 (CCN1) [63,64], procollagen I [63–65], MMP-1 [63,64,66], MMP-2 [67], and is able to down-regulate the accumulation of ECM. However, several independent studies found that RA could up-regulate the expression of FN [57,59], collagen I [59,60], collagen IV [68,69], TSP [57], LN [57,70], MMP-2 [71], MMP-9 [71], GPRC5B [72], integrin α5 [73], IGF1 [74] and IGF2 [74], which results in the opposite effect as reported above, *i.e*., the induction of ECM accumulation [68,75,76].

Interestingly, Varani *et al.* [77] found that at 0.5 μg/mL, ATRA stimulated the production of collagen I, but at higher concentrations (2.5 μg/mL), the production of collagen I was inhibited. Therefore, the effect of different doses of RA might have different results on cellular signaling and thus the extent to which constituents of signaling pathways are up- or down-regulated. In future research efforts, determining such dose-response curves would be paramount in order to both evaluate the true effect of RA on cell signaling and to determine the optimal dose for therapeutic interventions; either for RA itself or molecules that attenuate the effect of RA. Medium to high-throughput screening of multiple pathways in response to a wide RA concentration range seem to be inevitable. The signaling pathways for RA in ECM metabolism, based on our retrospective literature search and the current *status quo*, are summarized in Figure 1.

## 4. Conclusions and Perspectives

Because there was no review available that summed up the main pathways in which RA might play a role in the pathogenesis of fibrotic diseases, we performed an extensive search of the published literature and present an overview of the signaling pathways for RA. This review shows that RA not only effectively inhibits the expression of various Collagens, including Collagen III, Collagen 1A1, Collagen III, Procollagen I, but also a myriad of other biomolecules, such as α-SMA, TNF-α, and IL-6, *etc.*, and might therefore play a protective role in fibrotic disease, e.g., liver fibrosis, kidney fibrosis, *etc.* There might be a common pathway via which RA exerts this protective effect. However, since most indicators in the Figure 1 have not been confirmed over a variety of cells and organs, more studies are consequently required to corroborate this hypothesis. Equally important should be the search for a common pathway via which RA is able to exert adverse effects on normal physiological function and it is clear from Figure 1 that RA up-regulates a myriad of key regulators of cellular metabolism.

Our study also shows that the effect of RA on the extent of the fibrosis markedly varied between various studies we retrospectively examined. We speculate that there might be several possible reasons for the controversial role that RA plays in fibrotic diseases: (1) various doses of RA were used in different studies, which might directly be responsible for this controversy; (2) different isoforms of RA might induce different effects; (3) various tissue or cell types were used in various studies; (4) signaling pathway differences of different tissues/cells induce dissimilar results; (5) the timing of the experimental studies markedly affects the outcome, *i.e.*, early or late in fibrosis development.

Evidently, RA is involved in a myriad of signaling pathways, which take part in the pathogenesis of fibrotic diseases, and therefore the potential mechanisms that lead to its onset and progression are complicated. This leaves a turbid and incomplete picture of the exact role that RA plays in fibrosis and thus a significant effort to elucidate the mechanisms involved lies ahead. However, as more studies become available, especially using siRNA to selectively inhibit particular signaling pathways, this picture will certainly become clearer. Thus, comprehensive, focused and well controlled studies should be devised and performed in the near future.

## Figures and Tables

**Table 1:** Overview of retinoic acid isoform characteristics.

Name	IUPAC name	Molecular structure	Chemical formula	Molecular weight	Exp. Log *P*_ow_	ACD/Pred. Log *P*_ow_	Pharmaceutical classification
Tretinoin (all-*trans* retinoic acid)	(2*E*,4*E*,6*E*,8*E*)-3,7-dim ethyl-9-(2,6,6-trimethy l-1-cyclohexenyl)nona- 2,4,6,8-tetraenoic acid	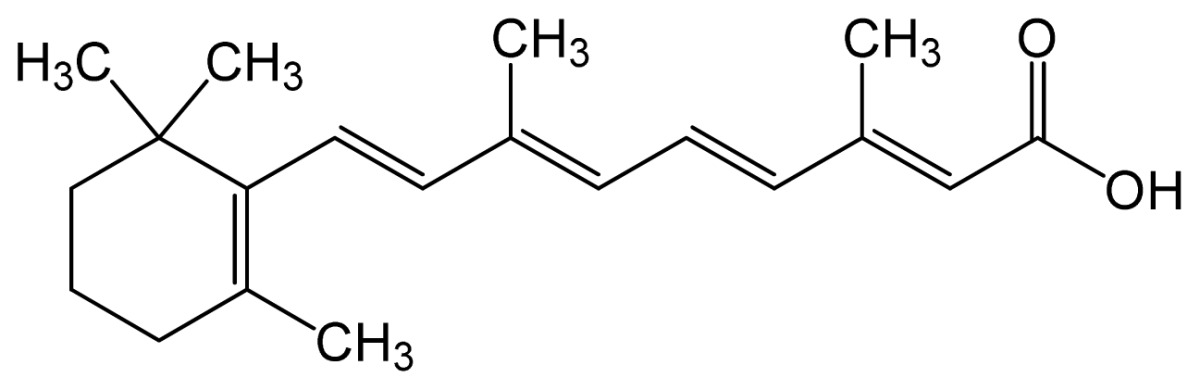	C_20_H_28_O_2_	300.44 (g/mol)	–	6.83	Antineoplastic Agents [D27.505.954.248] Keratolytic Agents [D27.505.954.444.400]
Alitretinoin (9-*cis* retinoic acid)	(2*E*,4*E*,6*Z*,8*E*)-3,7-dim ethyl-9-(2,6,6-trimethy lcyclohexen-1-yl)nona- 2,4,6,8-tetraenoic acid	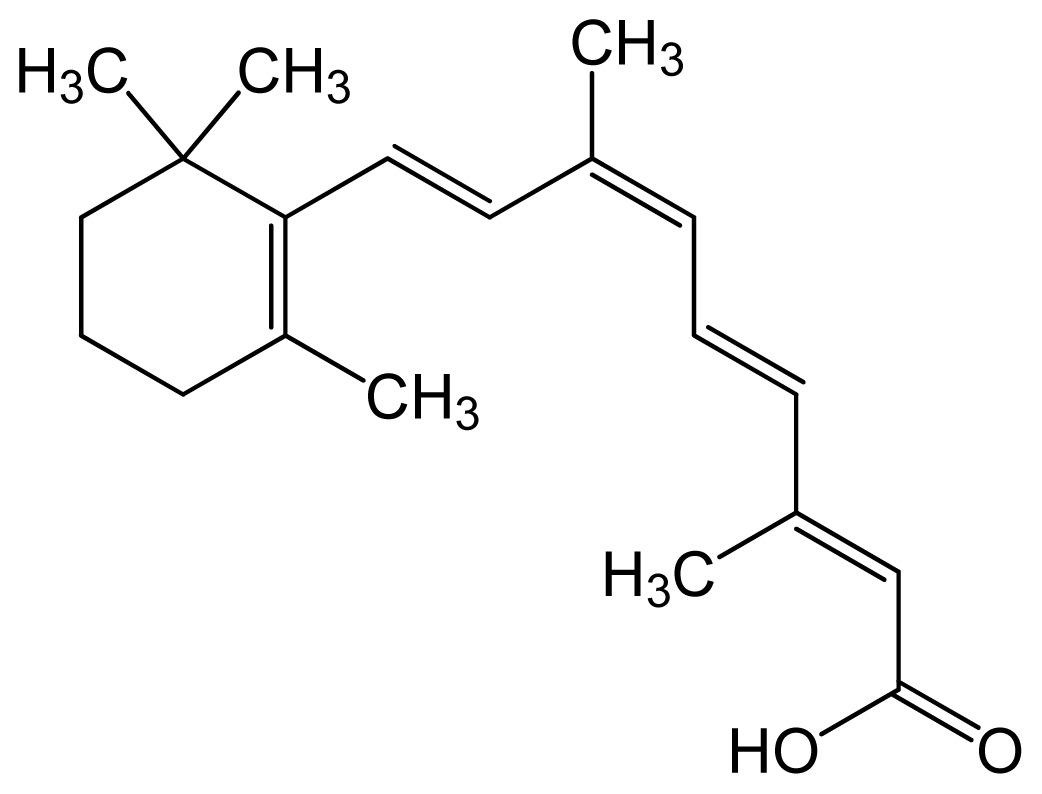			6.84	6.26	Panretin® gel: topical treatment of cutaneous lesions in patients with AIDS-related Kaposi’s sarcoma
Isotretinoin (13-*cis* retinoic acid)	(2*Z*,4*E*,6*E*,8*E*)-3,7-dim ethyl-9-(2,6,6-trimethy lcyclohexen-1-yl)nona- 2,4,6,8-tetraenoic acid	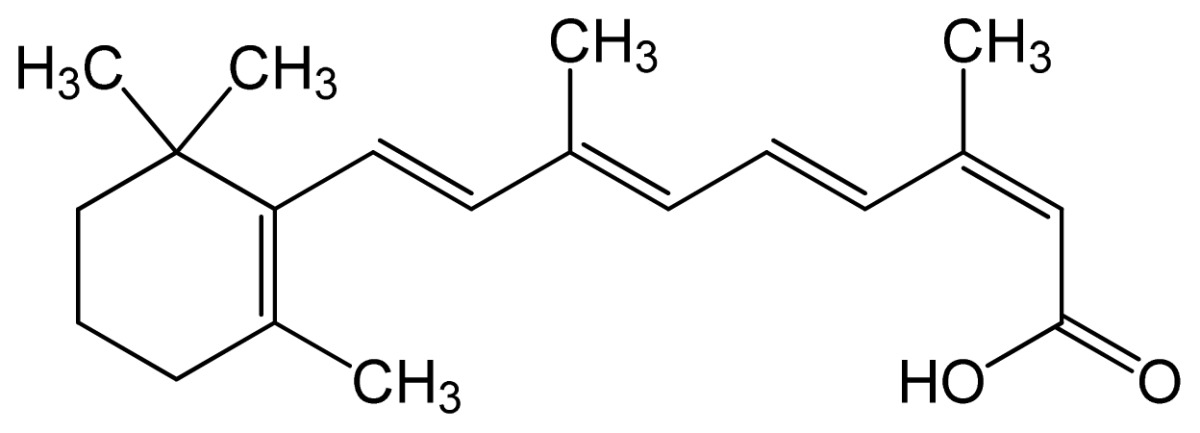			–	6.26	Topical dermatologic agent for treatment of *Acne Vulgaris* and several other skin diseases

Note: Log *P*ow represents the partition coefficient in octanol/water; ACD/Pred. = the predicted Log *P*ow with ACD software, based on the structural formula.

**Table 2:** Characteristics of the studies evaluating the effect of retinoic acid (RA) on liver fibrosis.

Author	Year	Type of animal/cells	Weight of animal/Age	Type of RA	Dose of RA	Effect
Hellemans *et al.*	1999	Hepatic stellate cells	–	ATRA or 9-*cis*-RA	0.01, 0.1, and 1 μM	+
Ye *et al.*	2010	Hepatic stellate cells	–	ATRA	0.01–10 μM	+
Wang *et al.*	2007	C57BL/6J mice	20–25 g	ATRA	1 mg/kg, three times per week	+
Yang *et al.*	2008	BALB/c mice	Eight-week-old	PL	30, 150 or 300 μg/kg.day	+
		Hepatic stellate cells	–	PL	2 ng/mL	+
Wang *et al.*	2008	Wistar rats	180–200 g	ATRA	0.1, 1.5 and 7.5 mg/kg, four consecutive weeks	+
He *et al.*	2011	Sprague-Dawley rats	200–230 g	ATRA	5 mg/kg, 15 consecutive days	+
		Hepatic stellate cells	–	ATRA	5 μM	+
Hisamori *et al.*	2008	BALB/c mice	Eight-week-old	ATRA	0.5 mg/mice, three times per week	+
		Hepatic stellate cells	–	ATRA	1 μM	+
Radaeva *et al.*	2007	Hepatic stellate cells	–	ATRA	NA	+
Okuno *et al.*	1997	Wistar rats	100–120g body weight	9-*cis*-RA	40 mg/kg, 5 times per week	−
		Hepatic stellate cells	–	9-*cis*-RA	0–10 μM	−

Note: RA = retinoic acid; ATRA = all-*trans* retinoic acid; PL = *P. linteus* mycelium, a retinoic acid derivative; NA = relative data were not available in original paper. Effect +: RA plays a protective role against liver fibrosis/extracellular matrix accumulation; Effect −: RA plays a negative role in liver fibrosis/induces the accumulation of extracellular matrix.

**Table 3:** Characteristics of the studies evaluating the effect of RA on pulmonary fibrosis.

Author	Year	Type of animal/cells	Weight of animal/Age	Type of RA	Dose of RA	Effect
Tabata *et al.*	2006	C57BL/6 mice	Eight-week-old	ATRA	0.5 mg/mice, repeated three times weekly	+
		Lung fibroblasts	–	ATRA	1 μM	+
Esteban-Pretel *et al.*	2010	Wistar rats	60-day-old	ATRA	100 μg/rat, 10 consecutive days	+
Ozer *et al.*	2005	Wistar rats	Postnatal day 3	ATRA	500 ug/kg, 10 consecutive days	+
Tabata *et al.*	2005	C57BL/6 mice	Eight-week-old	ATRA	0.5 mg/mice, repeated three times weekly	+
		Lung fibroblasts	–	ATRA	1 μM	+
Dong *et al.*	2012	C57BL/6 mice	18–22 g	ATRA	Repeated 3 times weekly, for 28 days	+

Note: RA = retinoic acid; ATRA = all-*trans* retinoic acid; Effect +: RA plays a protective role against pulmonary fibrosis/extracellular matrix accumulation.

**Table 4:** Characteristics of the studies evaluating the effect of RA on kidney fibrosis.

Author	Year	Type of animal/cells	Weight of animal/Age	Type of RA	Dose of RA	Effect
Wagner *et al.*	2000	Wistar rats	180–200 g	ATRA	10 mg/kg per day	+
Morath *et al.*	2001	Wistar rats	180–200 g	ATRA	10 mg/kg per day	+
Lehrke *et al.*	2002	Wistar rats	180–200 g	Ro-257386	80 mg/kg per day	+
Oseto *et al.*	2003	Wistar rats	Twelve-week-old	ATRA	30 mg/kg per day	+
Schaier *et al.*	2004	Wistar rats	145–150 g	AGN 195183 or AGN 194204	4 mg/kg, 20 mg/kg per day AGN 195183; 0.4 mg/kg,, 2 mg/kg per day AGN 194204	+
Adams *et al.*	2005	Rats	200–220 g	13-*cis*-RA	2 mg/kg per day	+
		Macrophages, Fibroblasts	–	13-*cis*-RA	10 μM	+
Wen *et al.*	2005	Glomerular mesangial cells	–	9-*cis*-RA	0.01–1 μM	+
He *et al.*	2007	Mice	–	ATRA	NA	+
		Podocytes	–	ATRA or 9-*cis*-RA	0.1–10 μM	+
Iyoda *et al.*	2007	Mice	–	ATRA	20 mg/kg per day	−
Xu *et al.*	2010	C57BL/6J × CBA F1 mice	One-week-old	ATRA	6–10.7 mg/kg per day; 12.7–18.8 mg/kg per day, 20.1–27.4 mg/kg per day	+
Liu *et al.*	2011	Sprague-Dawley rats	250–330 g	ATRA	5 mg/kg per day, 10mg/kg per day	+
Kishimoto *et al.*	2011	C57BL/B6 mice	25–30 g (Eight-week-old)	ATRA	20 mg/kg	+
Zhong *et al.*	2012	Mice	Four-week-old	Am580	0.3 mg/kg per day	+
Mallipattu *et al.*	2012	Podocytes	–	ATRA	1 μM	+
Moulder *et al.*	2002	Rats	–	ATRA	15 mg/kg per day	+
Zhou *et al.*	2011, 2012	Wistar rats	180–200 g	ATRA	15 mg/kg per day	+

Note: RA = retinoic acid; ATRA = all-*trans* retinoic acid; NA = relative data were not available in original paper. Effect +: RA plays a protective role against kidney fibrosis/extracellular matrix accumulation; Effect −: RA plays a negative role in kidney fibrosis/induces the accumulation of extracellular matrix.
